# Psychosocial changes during COVID-19 lockdown on nursing home residents, their relatives and clinical staff: a prospective observational study

**DOI:** 10.1186/s12877-023-03764-x

**Published:** 2023-02-03

**Authors:** Adriana Catarina De Souza Oliveira, María Gómez Gallego, Carmelo Gómez Martínez, Elena Carrasco Martínez, Jorge Moreno Molina, Juan José Hernández Morante, Paloma Echevarría Pérez

**Affiliations:** 1 Research Group of Nursing Languages in Social Context, Faculty of Nursing, UCAM Universidad Católica San Antonio de Murcia, Campus de Guadalupe, Murcia, Spain; 2 Department of Neurology and Mental Health, Faculty of Nursing, UCAM Universidad Católica San Antonio de Murcia, Murcia, Spain; 3 “Mensajeros de La Paz” Nursing Home Association, Murcia, Spain; 4 Eating Disorders Research Unit, UCAM Universidad Católica San Antonio de Murcia, Campus de Guadalupe, s/n, 30107 Murcia, Spain

**Keywords:** Lockdown, Nursing home, Depression, Anxiety, Humanization, Social support, Relatives, Staff

## Abstract

**Background:**

Previous works have observed an increase of depression and other psychological disorders on nursing home residents as a consequence of coronavirus disease 2019 (COVID-19) lockdown; however, there are few studies that have performed a comprehensive evaluation of all people involved in nursing homes environment. The objective of the work was to analyse the impact of lockdown on psychosocial factors of nursing home residents, relatives and clinical staff and how these variables have influenced residents’ survival.

**Methods:**

A prospective study was designed. Evaluations were performed at three different times: a) at the beginning of Spanish confinement, in March 2020; b) just before the second wave of the pandemic, with relaxation of security measures but in lockdown, and c) in January–February 2021, at the end of the second wave, when visits were already allowed. The study was conducted on three different nursing homes. Three hundred and one residents, 119 clinical staff and 51 relatives took part in the study. Anxiety and depression were evaluated in all participants. A scale on the meaning of suffering was also performed. In addition, burnout status was also determined in the clinical staff.

**Results:**

All participants showed lower depression during lockdown, while at the beginning and at the end of the confinement, these values were significantly increased. In residents, these changes were dependent of cognitive status (*p* = 0.012). Anxiety was significantly higher in residents. The evolution of anxiety was similar than with depression, with lower values during confinement, although clinical staff showed higher anxiety levels at the beginning. The feeling of suffering was significantly lower in the clinical staff than in resident and relative groups. Residents’ survival was dependent of cognitive status (*p* = 0.018) and voluntary confinement (*p* < 0.001).

**Conclusions:**

During the first COVID-19 lockdown, psychological wellbeing of residents cared in nursing homes, their relatives and staff did not seem to be seriously affected. Previous mental health in relatives and staff together with a resilient approach to the adversity might partly be protecting factors. The lack of consequences on residents’ anxiety, depression and perception of social support may reflect the special attention and care they received. Finally, as in the current study only data of the first two COVID-19 waves were analysed, its findings might be partly generalized to all the pandemic.

**Supplementary Information:**

The online version contains supplementary material available at 10.1186/s12877-023-03764-x.

## Background

The past years, the fast expansion of SARS_Cov2, the extension to the different population groups, independently of race, age, clinical and socio-sanitary characteristics, and the uncertain about clinical and therapeutic options to control this pandemic, yielded in an important public health problem [1]. As a consequence, coronavirus disease 2019 (COVID-19) triggered a world lockdown.

Social restrictions are health strategies usually implemented by governmental public health services to control and prevent public health problems [2]. The COVID-19 confinement has varied according to the epidemiological situation of each country. In Spain, the 14 March 2020, a state of alarm was declared, and a general confinement was applied [3]. In that date, European nursing homes for older people care where the health centre with higher mortality by COVID-19, with death rates over 50% of infected people [4]. Therefore, nursing homes were the first institutions implementing confinement measures, even mine if we take into account that older people were the most vulnerable population group to face with COVID-19, especially due to the presence of previous chronic diseases, polypharmacology and the own aging process [5].

As commented, the lockdown of nursing homes has been a health, psychosocial and mental health challenge, especially for the residents and their relatives, without forgetting the health professionals caring for the residents. In older adults, anxiety and depression are common psychological disorders, but they may have been enhanced by the pandemic and confinement [6]. Likewise, COVID-19 pandemic has been related to sleep disorders, distress and fear [7–10]. During the adaptation to lockdown, older adults in nursing homes have experienced fast changes in their habits. Consequently, feelings of suffering appear in response to confinement [11]. In addition, the communication breakdown with family members and with the social environment, may became a source of emotional loneliness and psychological distress [12]. On the other hand, relatives also suffered lockdown consequences. Relatives are an important support for older people; however, in the COVID-19 pandemic, there has been emotional exhaustion, which generated an imbalance in family’s coping process, which sometimes generates doubts about the role of the family as support or as a conflict in the care of adults [13].

Although previous works have evaluated the influence of lockdown on depression and other psychological features, no previous work have performed a comprehensive evaluation of all people involved in older adults’ care living in nursing homes. For this reason, the main objective of the present work was to prospective analyse the impact of lockdown on psychosocial factors of all players around nursing homes, residents, their relatives and clinical staff. As a secondary objective, we wanted to compare whether the evolution of these parameters was different between the different groups. Finally, we also wanted to study the influence of these variables on the residents’ survival.

## Methods

### Design and participants

To evaluate the changes on different psychological features during COVID-19 lockdown, a prospective observational study was designed. Evaluations were performed at three different times: a) at the beginning of Spanish confinement, in March 2020; b) just before the second wave of the pandemic, with relaxation of certain security measures but still in lockdown, in June–July 2020 and c) in January–February 2021, at the end of the second wave, when visits to nursing homes were already allowed.

The study was conducted on three different nursing homes of Murcia (South-East Spain). The three residents were private, and two were located in rural settings. Three hundred and one nursing home residents, 119 clinical staff and 51 relatives took part in the study. For staff and relatives, the only exclusion criterion was refusal to take part in the study. For residents, in addition, terminally ill residents and those with acute medical conditions that prevent the baseline evaluation (fractures, respiratory or other severe infections, heart attack, pneumonia, etc.) were also excluded. Patients with dementia and/or severe cognitive decline were also included, as representative of this population. In this case, permission was obtained from their legal guardians. Cognitive status and psycho-social features were evaluated by trained psychologist, social workers and geriatricians. Medical history was obtained from the medical records of the residents (a flow diagram of the study participants is available as Supplementary Fig. S1).

The present study was carried out after authorization of the Ethics Committee of the UCAM-Universidad Católica de Murcia. A Scientific Panel of the Nursing Homes also evaluated and approved the ethical characteristics of the present study. Oral and written permission was requested to staff, relatives and those residents with normal cognitive status. Those residents with marked cognitive impairment, permission was requested from their legal representatives.

### Measurements

In the present study, anxiety and depression were evaluated in residents, staff, and relatives. A scale on the perception about the meaning of suffering (evaluated with the “*Humanization questionnaire*”) was also performed in all participants. In addition, burnout status was also determined in the clinical staff, while social support was measured only in residents. All measurements were carried out by trained nurses, physiotherapist, psychologists and geriatricians with previous experience on nursing home work.

#### Anxiety

The Hamilton Anxiety Rating Scale (HARS) was used to evaluate anxiety status of the participants. It is 14-item scale hetero-administered by a clinician after an interview. The interviewer scores each item from 0 to 4 points. There are no cut-off points. A higher score indicates a greater intensity of anxiety [14]. The version used in the present study was the Spanish validation of the test, which was carried out by Lobo et al. in 2002 [15].

#### Depression

Depression was evaluated with two different tools, the Yesavage test, which was employed in the residents, and the Beck Depression Inventory-II (BDI-II) for clinical staff and relatives. The Yesavage test is a questionnaire specially designed for the depression screening in older adults (> 65 y) [16]. It is a test composed of 15 items, whose affirmative answers score 1 point (for a total of 15 points), in which the higher the score, the greater the degree of depression. The cut-off points are: 0–5: Normal, 6–10: Moderate depression, + 10: Severe depression. The Spanish version was validated by Martinez de la Iglesia et al. in 2002, showing a very high internal validity (alpha = 0.99) [17]. For clinical staff and relatives, BDI-II was conducted to screen depression. BDI-II is a more recent version of the classic 21 items test [18], which evaluates perceived depression in adults. Items are evaluated on a Likert scale (0–3 points), where higher scores indicate higher depression symptomatology. Although there are several cut-off points, a score higher than 13 points indicate mild depression [19]. The Spanish version of the test was validated previously [20].

#### Humanization suffering

This dimension was evaluated with a questionnaire (translated as “*Escala de Humanización*”) that evaluates the sense of suffering for a person. This 14-item scale is composed by two dimensions, suffering as a change, which indicates that the higher the sum of the scores obtained on these items, the more meaning or utility is given to suffering as a lever or engine of positive change. It can be as an experience of transcendence or as necessary energy that mobilizes a personal search and that leads to inner growth or a new life. The second dimension, suffering as a burden, indicates that the higher the sum of the scores obtained in these items, the more meaning is given to bearing the burden of suffering. The usefulness would be in the acceptance of the burden as sacrifice or effort, given the recognition that suffering is a consequence of irresponsible acts, bad actions committed, moral faults, desire, selfishness, or estrangement from the Divine. It also collects a sense of solidarity of acceptance of a shared burden with the world. It was originally developed in Spanish by Bermejo et al. [21]. The present study used the brief version (EHb), which was validated by the same group [22].

#### Social support

The DUKE-UNC questionnaire was performed on nursing home residents to evaluate the social support. The test score is a self-perception of social support, not the real one. It is an 11-item test with Likert-like responses. The score ranges from 11 to 55. The lower score, the lower support perception [23]. The Spanish version was previously validated, and a cut-off score of < 32 points was proposed as indicator of low perception of social support [24].

#### Burnout

The Maslach’s burnout inventory was employed to evaluate several features related with work overload in the clinical staff. This 22-item questionnaire evaluates three dimensions: *emotional exhaustion*, *personal accomplishment*, and *depersonalization*. Items are evaluated on a Liker scale [25]. The Spanish version has shown a good reliability and internal validity [26].

#### Other socio-demographic characteristics

Several independent variables such as age and sex were also registered. In addition, the days of stay on the nursing home, whether they received visits or not, or whether they decided on voluntary confinement or not, were also recorded for survival analysis.

### Statistical analysis

A basic descriptive analysis was conducted, estimating mean and 95% confidence interval for continuous variables and frequency and percentage for nominal variables. Normality was assessed by mean of the Kolmogorov-Smirnov test, confirming the normal distribution of continuous variables. Therefore, parametric tests were employed. Concretely, to analyse baseline differences regarding age and other psycho-social features, a one-way ANOVA was carried out, with a SIDAK’s post hoc test. This test was also employed to evaluate changes along time in those measurements carried out in only one group, as Yesavage or Burnout. Moreover, a mixed-effects general linear model, using time as within-subject factor and group as between-subject factor was also used to evaluate possible statistical differences along time in the different groups. To exclude a potential bias due to age, sex and baseline MMSE score, these variables were used as covariates. Finally, to evaluate a possible influence of the different psycho-social features on participant’s survival, an analysis was conducted according to the Kaplan-Meyer methodology. An additional Cox regression analysis was done to estimate the hazard ratio of these features on all-cause mortality. All analysis were performed with the SPSS 25.0 software (IBM SPSS, Chicago, IL). Significance value was set for all analysis at *p* < 0.05.

## Results

### Baseline characteristics of participants

Participants were mainly women (80%), with a similar sex distribution in the different groups (75% residents, 92% clinical staff and 80% of relatives being women). Supplementary Table S1 shows age and baseline psychological features of the participants. Age was statistically significant higher in the residents’ group, with an age that was twice of the clinical staff (*p* < 0.001). The anxiety perception, as indicated by Hamilton test, was algo significantly higher in residents (*p* = 0.001), while the ANOVA test revealed that there were no differences between relatives and clinical staff. The other features are described in Table S1.

### Evolution of depression on nursing homes during COVID-19 lockdown

**Fig. 1:**
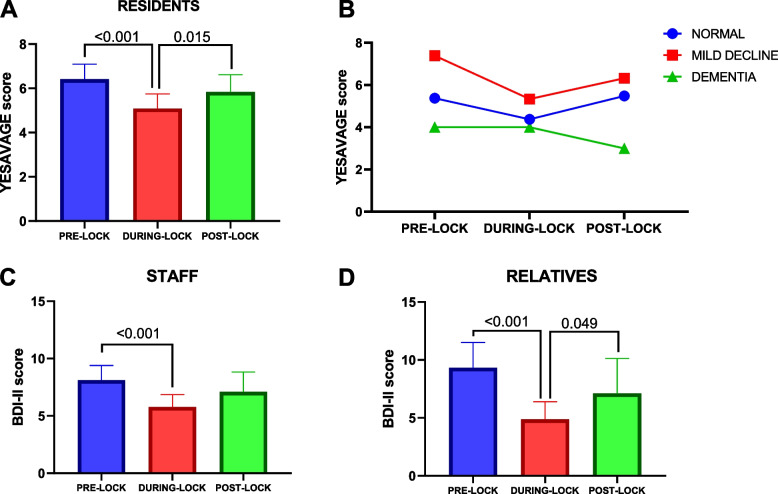
Evolution of depression status throughout study period. Figure represents changes on Yesavage Geriatric Depression Scale (YGDS) along study period in nursing home residents (**A**) and in the same group attending to their baseline cognitive status (**B**). Lower panel (**C** and **D**) represents changes on Beck’s Depression Inventory-II Scale along study period in clinical staff (**C**) and relatives (**D**). Data represent mean ± sd. Statistical differences were evaluated by a repeated measures general linear model. Post hoc evaluation of differences among time periods were performed with Sidak’s test for multiple comparison (For YGDS: *F* = 16,900, gl = 98, partial η^2^ = 0.256. For staff: *F* = 3.935, gl = 70, partial η^2^ = 0.101; For relatives: *F* = 24.493, gl = 40, partial η^2^ = 0.550)

On the residents, the statistical analysis showed a significant decrease of depression scores from the beginning of the confinement to the second evaluation time, which indicates less self-perception of depression in this period, but this situation was reversed at the end of the quarantine (Fig. 1A). This data was independent of sex (*p* = 0.248), however, there was an interaction with participants’ age (*p* = 0.032), in the sense that older subjects showed a greater increase of depression at the end of the study period, which indicates that older subjects were more sensitive to lockdown in terms of depression. At the same time, changes on Yesavage scale were also dependent of cognitive status (*p* = 0.012) since those patients with normal cognitive status and mild cognitive decline showed an increase of the perception of depression at post-lockdown evaluation (Fig. 1B).

Figure 1 shows depression scores on the clinical staff (Fig. 1C) and family relatives (Fig. 1D). As with residents, the higher depression status was observed at baseline. Again, a statistically significant reduction of depression symptomatology was observed during the lockdown in both staff and relatives, namely, lockdown reduced the depression status of these participants; however, at the end of the study, this symptomatology increased, but in this occasion, statistically significance was only observed in family and relatives, which indicates that the depression status increase was more moderate in clinical staff. In this time, age and sex were not associated with staff or relatives depression score (*p* > 0.050 in all cases).

### Evolution of anxiety on nursing homes during COVID-19 lockdown

**Fig. 2:**
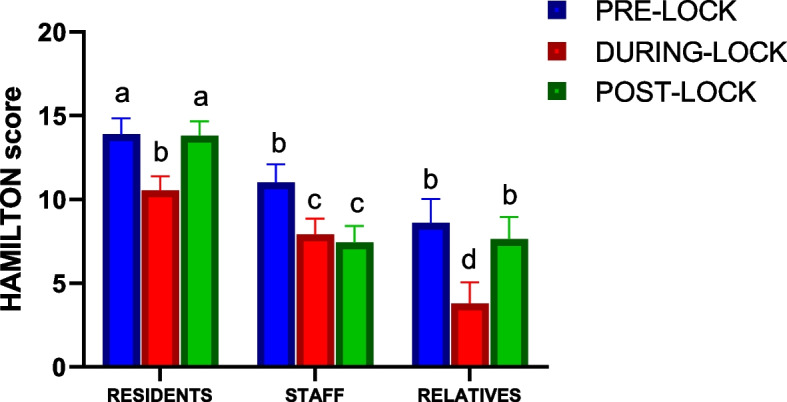
Evolution of anxiety status throughout study period. Figure represents changes on Hamilton Anxiety Rating Scale along study period in all groups. Data represent mean ± sd. Statistical differences were evaluated by a repeated measures general linear model. Post hoc evaluation of differences among time periods were performed with Sidak’s test for multiple comparison. Different letters represent statistically significant differences (*F* = 25.065, gl = 213, partial η^2^ = 0.191)

The data revealed significant higher anxiety level in residents compared to clinical staff (*p* = 0.002) and relatives (*p* < 0.001), who showed the lower anxiety intensity (Fig. 2). The course of the disease was different depending on the group. Residents showed a significant decrease of anxiety during lockdown, although at the end of the confinement, the anxiety level came back to baseline levels. The clinical staff showed higher anxiety levels at the beginning of the confinement, although there was a significant reduction throughout the study period. On the other hand, relatives had a similar behaviour regarding anxiety that residents, since they also had a significant reduction of their perception of anxiety during lockdown, which was increased to similar values that at the beginning of the confinement. These differences were also confirmed by the significant interaction between anxiety and group (*p* = 0.032) revealed by the statistical analysis. Similarly, sex also interacted with anxiety scores along time (*p* = 0.029). In fact, men tended to show a similar anxiety score through the study, while women were characterized by a clear increase post-lockdown; however, covariate analysis did not show any interaction between HARS and age (*p* = 0.467).

### Evolution of humanization suffering on nursing homes during COVID-19 lockdown

The pandemic has led to a large number of deaths, which has obviously been associated with an increased feeling of suffering. For this reason, in the present work we evaluated the perception of people associated with the environment of nursing homes through the humanization scale (Supplementary Fig. S2). Regarding the feeling of suffering as a change (Fig. S2-A), within every group, there were no statistically significant differences at any time evaluated. However, it is interesting to highlight that this feeling was significantly lower in the clinical staff than in resident and relative groups. Similarly, the “suffering as a burden” dimension showed a similar trend (Fig. S2-B), without changes throughout the confinement but being always lower in clinical staff.

### Evolution of functional social support on nursing home residents

Attending to data obtained, the nursing home residents had a similar perception about their social support throughout the study period (*p* = 0.219) (Fig. S3). In addition, this perception was independent of sex (*p* = 0.329), age (*p* = 0.440) or baseline cognitive status (*p* = 0.279).

### Evolution of burnout on nursing homes clinical staff

There were no statistically significant changes during the COVID-19 lockdown in the clinical staff for exhaustion and depersonalization variables (Fig. S4). Nevertheless, exhaustion was increased at the end of the confinement, as occurred with depersonalisation dimension. In contrast, personal accomplishment was statistically significant decreased at the end of the lockdown. Covariate analysis revealed a significant interaction for exhaustion and sex (*p* = 0.044), mainly because in men, there was a marked decrease of exhaustion at post-lockdown, while in women, the situation was just the opposite. There were no other statistically significant interactions between burnout variables and sex or age (*p* > 0.050 in all cases).

### Influence of psychological features on residents’ survival during lockdown

To determine the influence of independent socio-psychological features on residents’ survival, the Kaplan-Meyer analysis was conducted. Attending to this data, survival was independent of the residents’ perception of anxiety (*p* = 0.426) social support (*p* = 0.347) and depression (*p* = 0.628). Similarly, we did not observe differences in survival depending on whether the patients received visits from their relatives during confinement (*p* = 0.112).

**Fig. 3:**
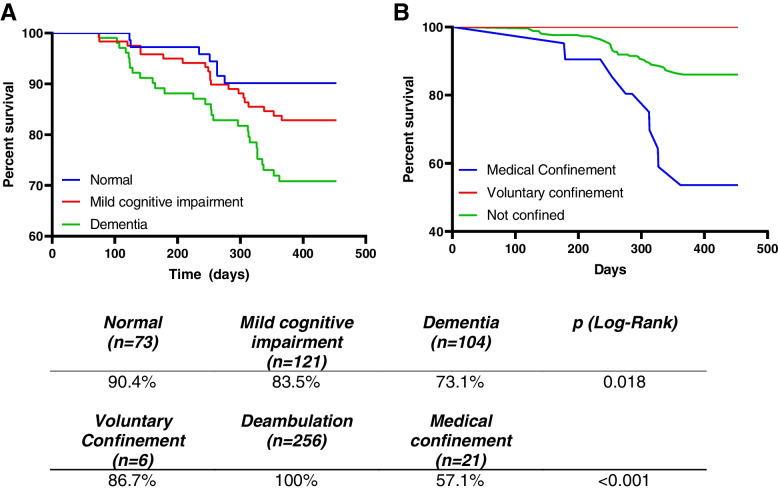
Kaplan–Meier Curves for the different groups attending to their (**A**) cognitive status or (**B**) the type of confinement during covid lockdown. The horizontal axis refers to the time in number of days since lockdown

An interesting data from the study was related to the differences in mortality depending on whether the residents decided a voluntary confinement, were confined by medical conditions, or freely moved in the nursing homes. Concretely, mortality was statistically significantly lower in those in voluntary confinement (log-rank chi = 17.466, *p* < 0.001). Survival was also associated with cognitive status, since those with normal status showed higher survival rate (log-rank chi = 8.059, *p* = 0.018) (Fig. 3).

## Discussion

The present study aimed to evaluate the evolution of certain socio-psychological characteristics during COVID-19 confinement in all nursing home members, residents, clinical staff and family and relatives. According to our data, the nursing homes played a protective role during confinement since a reduction in the depression and anxiety statuses in residents, clinical staff and family members was observed. Interestingly, some of these features returned to their baseline values when the lockdown ended, and visits were allowed again. In contrast to some previous studies [27, 28], our data do not suggest that lockdown in nursing homes had negative effects on residents’ mental health. It is tempting to speculate that a more peaceful environment caused by social restrictions might contribute to this finding. In addition, as Mc Arthur et al. reported [29], ongoing monitoring and periodic evaluation of residents’ mental health might have allowed to implement effective care and treatment that mitigates the lockdown negative effects. As a matter of fact, social restrictions derived from lockdown were not found to have consequences on residents’ perception of social support. Perhaps, a greater effort of nursing homes staff helped to supply the lack of visits by their relatives. In fact, in older adults, having basic needs met is associated to greater psychological wellbeing [29]. Not surprisingly, we did not find any impact of perceived social support, be visited by relatives and depression on residents’ survival. Instead, dementia was found to be an important factor associated to shorter survival, as other found [30]. One reason might be that these patients are less likely to understand social restrictions due to the pandemic and suffer higher apathy, irritability, or anxiety [31].

In a similar way, relatives did not show either higher anxiety or depression throughout the COVID-19 lockdown. Even, these scores decreased at the end of the first lockdown. This time many of the restrictions had been lifted because the risk of COVID-19 infection was lower. Visiting restrictions may impact negatively on those who continue to provide personal care to relatives after institutionalization. This lack of interaction may cause on relatives a feeling of loneliness due to having greater concern for the health of their relatives residing in the nursing homes [32], as well as more anxiety and depressive symptoms [33]. Another risk factor of low psychological wellbeing is to have previous psychiatric disorders and neuroticism [34]. In the current study, relatives presented low baseline scores of anxiety and depression that might protect their mental health [35, 36].

Our results about staff wellbeing are in contrast to previously suggested by cross-sectional studies [37, 38]. In this study, staff did not show and increase in depressive symptoms during lockdown. Moreover, anxiety levels were even lower at the end of the second wave than at the beginning of the lockdown. Likewise, the scores of emotional exhaustion, depersonalization and personal accomplishment indicated low burnout [39].

As happened to relatives, before lockdown mental health of staff might not be disturbed, as shown baselines scores, leading to a lower risk to present psychological symptoms [40]. Another potential reason for these findings was a higher resilience of staff. Nursing homes lockdown involved important restrictions in social relationships, high doses of exposure to suffering and an increased workload for nursing home staff [41]. Prolonged exposition to these stressors is likely to have negative effects on health [42]. Resilience is understood as an ability to adapt successfully in stressful situations and adversity [43]. In fact, during the pandemic health workers identify resilience as powerful tool to preserve mental health [44]. Although in this work resilience was not assessed, the lack of higher burnout across time suggest they developed a highly adaptative strategy to face the adversity. In addition COVID-19 pandemic not only had undesirable effects on the population health and economy but also brought greater social and professional appreciation of the healthcare workers together with a perspective of higher employability [45]. This situation might have been key to maintain staff mental health and prevent burden [46].

Finally, we must highlight that certain parallelism in the changes of depression scores between residents, relatives and staff across time was observed. Such finding is in agreement with literature concerning the association between residents care and wellbeing and staff burnout [47].

At this point, several caveats should be commented. Firstly, although the present work has a similar sample size that previous works, we still believe that it is a relatively small sample to extrapolate the observations to the general population. In addition, although the present work is a three-centre study, all nursing homes were in a specific geographical region, which again limits the extrapolation of the data. On the other hand, although the measurement instruments employed have been psychometrically validated and have also been widely used in the literature, the dimensions evaluated such as social support, feelings of anxiety or depression sometimes deserve a qualitative approach, which would have helped to interpret the data obtained. On the other hand, the longitudinal design and the use of validated tools have allowed us to evaluate the impact of confinement due to COVID-19.

In conclusion, during the lockdown at the first two COVID-19 waves, psychological wellbeing of residents cared in nursing homes, their relatives and staff did not seem to be seriously affected. Previous mental health in relatives and staff together with a resilient approach to the adversity might partly be protecting factors. The lack of consequences on residents’ anxiety, depression and perception of social support may reflect the special attention and care they received. In any case, we cannot rule out certain worsening of mental health in subjects in relation to pandemic as no measures was collected just before lockdown. Finally, as in the current study only data of the first two waves were analysed, its findings might be partly generalized to all the pandemic.

## Supplementary Information

**Additional file 1: Table S1.** Baseline characteristics of study participants. **Supplementary Fig. S1.** Study flow diagram. **Supplementary Fig. S2.** Evolution of Humanization suffering perception throughout study period. Figure represents changes on “suffering as a change” (A) and “suffering as a burden” (B) scales of the Humanization Scale along study period in all groups. Data represent mean ± sd. Statistical differences were evaluated by a repeated measures general linear model. Post hoc evaluation of differences among time periods were performed with Sidak’s test for multiple comparison. Different characters represent statistically significant differences (For suffering as a change: *F* = 0.965, gl = 208, partial η^2^ = 0.009; For suffering as a burden: *F* = 0.610, gl = 208, partial η^2^ = 0.006). **Supplementary Fig. S3.** Evolution of social support perception in nursing home residents during study period. Data refer to Figure represents changes on DUKE-UNC-11 score in nursing home residents. Data represent mean ± sd. Statistical differences were evaluated by a repeated measures general linear model. Post hoc evaluation of differences among time periods were performed with Sidak’s test for multiple comparison. There were no statistically significant differences along time (*F* = 1.542, gl = 104, partial η^2^ = 0.029). **Supplementary Fig. S4.** Evolution of burnout status in clinical staff during study period. Figure represents changes on (A) exhaustion, (B) depersonalization and (C) personal accomplishment dimensions of Maslach burnout scale along study period in clinical staff. Data represent mean ± sd. Statistical differences were evaluated by a repeated measures general linear model. Post hoc evaluation of differences among time periods were performed with Sidak’s test for multiple comparison (For exhaustion: *F* = 0.990, gl = 71, partial η^2^ = 0.027; For depersonalization: *F* = 0.564, gl = 71, partial η2 = 0.016; For personal accomplishment: *F* = 8.975, gl = 71, partial η^2^ = 0.202).

## Data Availability

The datasets generated and/or analysed during the current study are available in the Mendeley repositor [https://data.mendeley.com/datasets/7pr8b8j92m/1].
