# Supplementary material for: Psychosocial changes during COVID-19 lockdown on nursing home residents, their relatives and clinical staff: a prospective observational study
Source: BMC Geriatr. 2023 Feb 3;23:71. doi: 10.1186/s12877-023-03764-x (PMC9897885; doi:10.1186/s12877-023-03764-x)

SUPPLEMENTARY INFORMATION

Table S1. Baseline characteristics of study participants

	RESIDENTS (n=301)	CLINICAL STAFF (119)	RELATIVES (n=51)
Age (y)	85 (84-86)	41 (39-43)	59 (56-62)
HARS	14.8 (13.0-16.5)	11.0 (8.9-13.0)	9.3(7.2-11.4)
Yesavage	6.4 (5.7-7.1)	-	-
BDI-II	-	8.1 (6.8-9.4)	9.3 (7.2-11.5)
Suffering as a change	23.7 (22.7-24.7)	21.4 (20.1-22.8)	24.5(23.3-25.7)
Suffering as a burden	15.6 (14.7-16.4)	12.1 (11.2-12.9)	14.3 (13.4-15.3)
DUKE	36.0 (34.3 – 37.8)	-	-
Exhaustion	-	13.9 (12.3-15.4)	-
Depersonalization	-	4.8 (4.1-5.6)	-
Personal accomplishment	-	34.5 (32.8-36.2)	-

Data represent mean and 95% confidence interval between parentheses. Excepting age, the other variables are shown as test scores. HARS: Hamilton Anxiety Rating Scale. Yesavage: Geriatric Depression Scale of Yessavage. BDI-II. Beck's Depression Inventory-II. DUKE: duke-unc-11 social support index. Suffering as a change and suffering as a burden, , Exhaustion, Depersonalization and Personal accomplishment

Supplementary Figure S1. Study flow diagram.

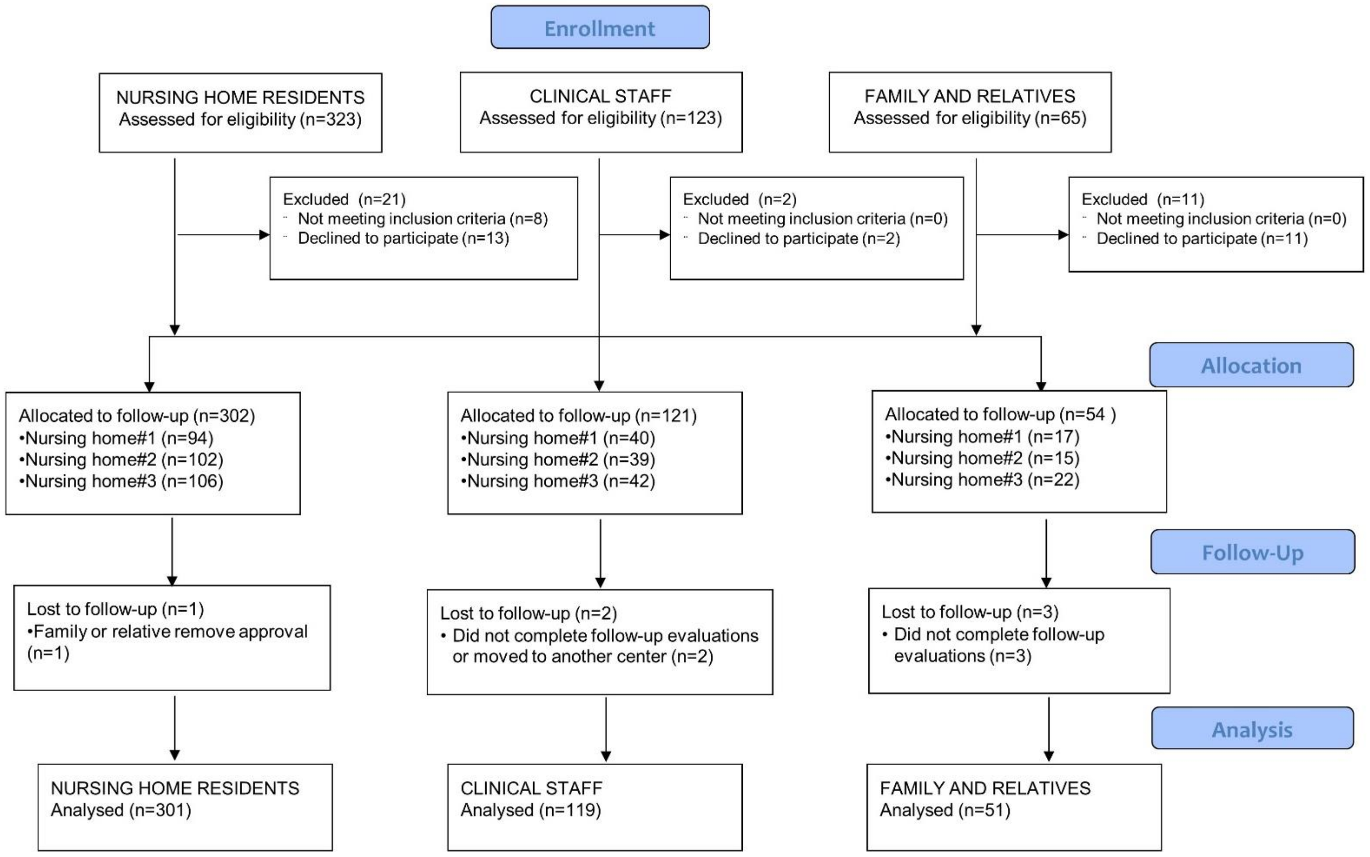

Supplementary Figure S2. Evolution of Humanization suffering perception throughout study period. Figure represents changes on “suffering as a change” (A) and “suffering as a burden” (B) scales of the Humanization Scale along study period in all groups. Data represent mean ± sd. Statistical differences were evaluated by a repeated measures general linear model. *Post hoc* evaluation of differences among time periods were performed with Sidak’s test for multiple comparison. Different characters represent statistically significant differences (For suffering as a change: $F = 0.965$, gl= 208, partial $\eta^2 = 0.009$; For suffering as a burden: $F = 0.610$, gl= 208, partial $\eta^2 = 0.006$).

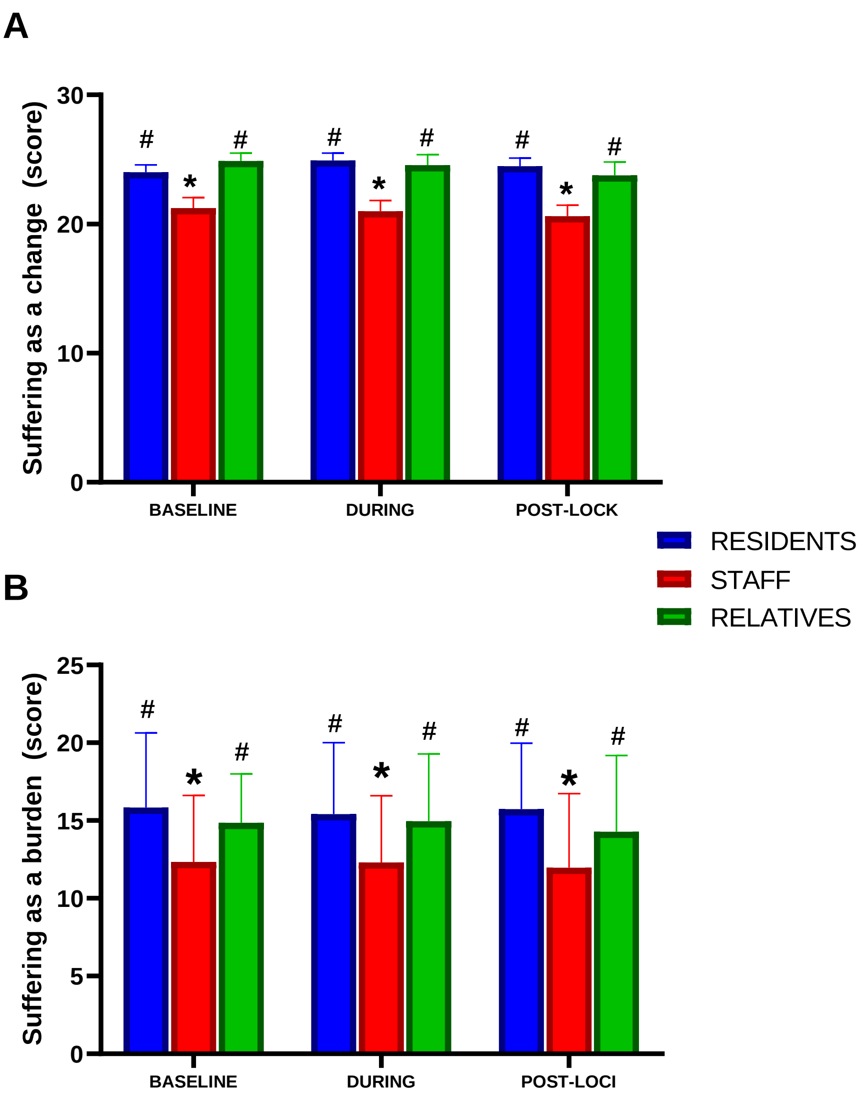

Supplementary Figure S3. Evolution of social support perception in nursing home residents during study period. Data refer to Figure represents changes on DUKE-UNC-11 score in nursing home residents. Data represent mean ± sd. Statistical differences were evaluated by a repeated measures general linear model. *Post hoc* evaluation of differences among time periods were performed with Sidak's test for multiple comparison. There were no statistically significant differences along time (F = 1.542, gl= 104, partial η^2 =0.029).

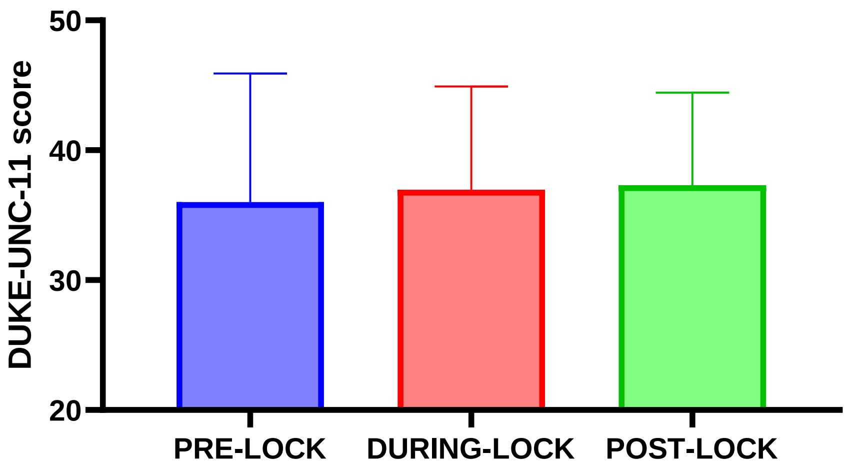

Supplementary Figure S4. Evolution of burnout status in clinical staff during study period. Figure represents changes on (A) exhaustion, (B) depersonalization and (C) personal accomplishment dimensions of Maslach burnout scale along study period in clinical staff. Data represent mean ± sd. Statistical differences were evaluated by a repeated measures general linear model. *Post hoc* evaluation of differences among time periods were performed with Sidak's test for multiple comparison (For exhaustion: F = 0.990, gl= 71, partial η^2 =0.027; For depersonalization: F = 0.564, gl= 71, partial η^2 =0.016; For personal accomplishment: F = 8.975, gl= 71, partial η^2 =0.202).

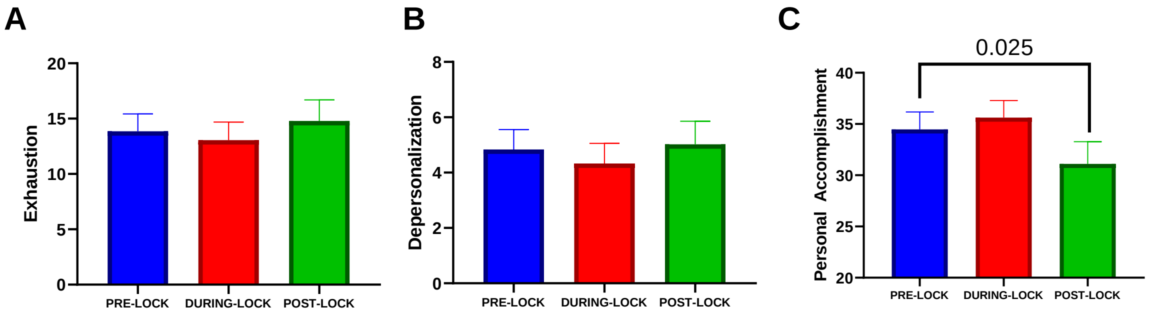

Supplement: Supplementary file 1 — Additional file 1: Table S1. Baseline characteristics of study participants. Supplementary Fig. S1. Study flow diagram. Supplementary Fig. S2. Evolution of Humanization suffering perception throughout study period. Figure represents changes on “suffering as a change” (A) and “suffering as a burden” (B) scales of the Humanization Scale along study period in all groups. Data represent mean ± sd. Statistical differences were evaluated by a repeated measures general linear model. Post hoc evaluation of differences among time periods were performed with Sidak’s test for multiple comparison. Different characters represent statistically significant differences (For suffering as a change: F = 0.965, gl = 208, partial η2 = 0.009; For suffering as a burden: F = 0.610, gl = 208, partial η2 = 0.006). Supplementary Fig. S3. Evolution of social support perception in nursing home residents during study period. Data refer to Figure represents changes on DUKE-UNC-11 score in nursing home residents. Data represent mean ± sd. Statistical differences were evaluated by a repeated measures general linear model. Post hoc evaluation of differences among time periods were performed with Sidak’s test for multiple comparison. There were no statistically significant differences along time (F = 1.542, gl = 104, partial η2 = 0.029). Supplementary Fig. S4. Evolution of burnout status in clinical staff during study period. Figure represents changes on (A) exhaustion, (B) depersonalization and (C) personal accomplishment dimensions of Maslach burnout scale along study period in clinical staff. Data represent mean ± sd. Statistical differences were evaluated by a repeated measures general linear model. Post hoc evaluation of differences among time periods were performed with Sidak’s test for multiple comparison (For exhaustion: F = 0.990, gl = 71, partial η2 = 0.027; For depersonalization: F = 0.564, gl = 71, partial η2 = 0.016; For personal accomplishment: F = 8.975, gl = 71, partial η2 = 0.202). [file 12877_2023_3764_MOESM1_ESM.pdf]
